# Outpatient Parenteral Antimicrobial Therapy for *Pseudomonas aeruginosa* Infections: Effectiveness and Safety

**DOI:** 10.3390/pharmaceutics18050549

**Published:** 2026-04-29

**Authors:** Paloma Suárez-Casillas, Marta Mejías-Trueba, Iris Martínez Alemany, Lola Navarro Amuedo, Julia Praena Segovia, Arístides de Alarcón González, Rafael Luque Márquez, Zaira R. Palacios-Baena, Juan Manuel Carmona-Caballero, José Manuel Sánchez Oliva, María Victoria Gil-Navarro, Manuel García Gutiérrez, Laura Herrera-Hidalgo, Manuel Poyato Borrego, Luis E. López-Cortés, José M. Cisneros

**Affiliations:** 1 Unidad de Gestión Clínica de Farmacia, Instituto de Biomedicina de Sevilla (IBiS), Hospital Universitario Virgen del Rocío/CSIC/Universidad de Sevilla, 41013 Seville, Spain; palomasuacas@gmail.com (P.S.-C.); martamejiastrueba@hotmail.com (M.M.-T.); mariav.gil.sspa@juntadeandalucia.es (M.V.G.-N.); 2 Unidad Clínica de Enfermedades Infecciosas, Microbiología y Parasitología (UCEIMP), Instituto de Biomedicina de Sevilla (IBiS), Hospital Universitario Virgen del Rocío/CSIC/Universidad de Sevilla, 41013 Seville, Spain; lola_navarro_amuedo@hotmail.com (L.N.A.); juliapraena@gmail.com (J.P.S.); aa2406ge@yahoo.es (A.d.A.G.); rafaeluquemarquez@gmail.com (R.L.M.); juanmacarmona81@gmail.com (J.M.C.-C.); josem.sanchez.oliva.sspa@juntadeandalucia.es (J.M.S.O.); manu162342@gmail.com (M.G.G.); jmcisnerosh@gmail.com (J.M.C.); 3 Centro de Investigación en Red de Enfermedades Infecciosas (CIBERINFEC), Instituto de Salud Carlos III, 28029 Madrid, Spain; zaira.palacios.baena@hotmail.com (Z.R.P.-B.); luiselopezcortes@gmail.com (L.E.L.-C.); 4 Unidad de Gestión Clínica de Enfermedades Infecciosas y Microbiología, Hospital Universitario Virgen Macarena, 41004 Seville, Spain; alemanyiris@gmail.com

**Keywords:** outpatient parenteral antimicrobial therapy, *Pseudomonas aeruginosa*, effectiveness, safety

## Abstract

**Objective:** Given the increasing use of outpatient parenteral antimicrobial therapy (OPAT) and the clinical challenges posed by *Pseudomonas aeruginosa* infections, this study aimed to evaluate the effectiveness and safety of OPAT for the treatment of *P. aeruginosa* infections in a real-world cohort. **Methods:** We conducted a prospective observational study with retrospective analysis including adult patients with *P. aeruginosa* infections treated within a multidisciplinary OPAT program shared by two tertiary hospitals between November 2012 and December 2024. Clinical characteristics, infection type, antimicrobial therapy, resistance patterns, source control, and clinical outcomes were recorded. Primary outcomes were treatment failure during OPAT and within 30 days after OPAT completion. Secondary outcomes included adverse events and vascular access complications. Bivariate and multivariable logistic regression analyses were performed to identify factors associated with treatment failure. **Results:** A total of 290 patients were included. The most frequent infections were bronchiectasis exacerbations (39.7%) and complicated urinary tract infections (15.2%). Most patients received monotherapy (72.8%), mainly ceftazidime, while 27.2% received combination therapy with a beta-lactam plus an aminoglycoside. Treatment failure occurred in 7.6% of patients during OPAT and in 15.5% within 30 days after OPAT completion, with an overall clinical success rate of 77%. Male sex and chronic obstructive pulmonary disease (COPD) were independently associated with failure during OPAT. At 30 days, higher Charlson comorbidity index, COPD exacerbation, and endovascular infection were associated with failure, whereas combination therapy was associated with a lower risk of failure. Antimicrobial-related adverse events were rare (3.2%). **Conclusions:** Our results support OPAT as an effective and safe strategy for managing *P. aeruginosa* infections in clinically stable patients. Patients with COPD, either as a comorbidity or during an exacerbation, and those with a higher Charlson score may require closer follow-up.

## 1. Introduction

Outpatient Parenteral Antimicrobial Therapy (OPAT) is a therapeutic approach that enables clinically stable patients to receive intravenous antimicrobial agents in non-hospital settings. This model of care is supported by a multidisciplinary team, typically involving infectious disease physicians, pharmacists, and specialized nursing staff. OPAT has demonstrated multiple advantages, including reduced length of hospital stay, decreased healthcare expenditures, and improved patient quality of life. It also facilitates a quicker return to daily activities [1,2,3]. Despite these advantages, OPAT faces ongoing challenges, including limited resources in certain regions, unplanned medical care visits, antimicrobial stability issues and the need for close monitoring to promptly detect potential complications [2,4].

OPAT success mainly relies on patient selection, along with the type of infection and the pathogen involved. *Pseudomonas aeruginosa* is a Gram-negative, non-fermenting, aerobic bacterium with intrinsic resistance mechanisms, including efflux pumps, beta-lactamases, and reduced membrane permeability, which make it particularly difficult to treat. It commonly causes serious hospital-acquired infections such as ventilator-associated pneumonia, bloodstream infections, and urinary tract infections, especially in immunocompromised individuals [5,6]. These infections, with high mortality rates and frequent recurrences, have limited oral treatment options and often require prolonged, high-dose intravenous antimicrobial therapy, in accordance with EUCAST dosing recommendations, accompanied by close clinical monitoring, thereby hindering the feasibility of outpatient care [7].

First-line agents against *P. aeruginosa* typically include ureidopenicillins (e.g., piperacillin-tazobactam), third- and fourth-generation cephalosporins (e.g., ceftazidime and cefepime), carbapenems (e.g., meropenem and imipenem) and fluoroquinolones (e.g., ciprofloxacin and levofloxacin) [6]. However, the global rise of multidrug-resistant (MDR), extensively drug-resistant (XDR), and carbapenem-resistant (CR) strains has markedly limited therapeutic options. To improve clinical decision-making, the concept of difficult-to-treat resistance (DTR) has been introduced. DTR refers to bacteria that are resistant to all standard first-line antibiotics with proven efficacy and manageable toxicity, including aztreonam, ceftazidime, cefepime, ciprofloxacin, imipenem-cilastatin, levofloxacin, meropenem, and piperacillin-tazobactam. The therapeutic arsenal against DTR isolates include antimicrobials developed in recent decades such as ceftolozane-tazobactam, ceftazidime-avibactam, imipenem-relebactam, and cefiderocol. This classification provides a more clinically relevant assessment of resistance compared to traditional MDR/XDR/CR definitions, as it reflects the absence of safe and effective standard treatments [8,9].

Given these complexities, managing *P. aeruginosa* infections in the outpatient setting is particularly challenging [6,8]. Although OPAT has been successfully reported in small cohorts involving *P. aeruginosa* infections, the overall body of evidence remains limited [10,11,12,13]. Further research is warranted to determine whether OPAT represents an appropriate and effective therapeutic strategy for infections caused by *P. aeruginosa*. In this context, the present study aimed to evaluate the effectiveness and safety of OPAT in a cohort of patients with *P. aeruginosa* infections.

## 2. Material and Methods

### 2.1. Study Design and Setting

A prospective observational study with retrospective analysis was conducted in a cohort of adult patients with *P. aeruginosa* infections treated within DOMUS, an OPAT program shared by two tertiary care hospitals in the same city. The OPAT program, previously described in the literature, is designed for clinically stable patients requiring prolonged intravenous antimicrobial therapy [1]. Inclusion and antimicrobial treatment decisions were made by a multidisciplinary team based on clinical criteria.

The program included home visits by a specialized nursing team for drug administration and monitoring, alongside weekly evaluations by an infectious disease specialist. All antimicrobials were prepared under sterile conditions by the hospital pharmacy service. Antibiotic stability was assessed prior to OPAT inclusion and approved by the multidisciplinary OPAT team. All antibiotics used in this study were stable for at least 24 h at room temperature in 0.9% sodium chloride, based on previously published data. The use of electronic infusion pumps (not in direct contact with the patient’s body) allowed the application of these stability data in the OPAT setting. Patients were instructed to maintain appropriate storage conditions, reinforced during nursing follow-up visit. Microbiological follow-up was performed according to the type of infection as part of routine clinical practice.

This study was conducted and reported in accordance with the STROBE guidelines [14]. The program was approved by the Ethics Committee for Clinical Research of both participating hospitals.

### 2.2. Patient Population

Inclusion criteria comprised all adult patients diagnosed with *P. aeruginosa* infections who were enrolled in the DOMUS OPAT programme between November 2012 and December 2024. Patients receiving OPAT for palliative care in an end-of-life context or those with polymicrobial infections were excluded. In patients with more than one *P. aeruginosa* infection, only those occurring more than 30 days after the initial infection were analysed as new episodes of infection.

### 2.3. Data Collection and Definitions

The following data were collected: sex, age, Charlson comorbidity index (CCI), specific comorbidities, infection diagnosis, control of the source of infection, date of initiation in DOMUS. Susceptibility testing was recorded, and isolates were classified as DTR (resistance to aztreonam, ceftazidime, cefepime, ciprofloxacin, imipenem-cilastatin, levofloxacin, meropenem, and piperacillin-tazobactam) [8]. Antimicrobial treatment (inpatient and outpatient) categorized as: combination therapy (beta-lactams plus aminoglycosides), and monotherapy, including ceftazidime, piperacillin-tazobactam, meropenem, other beta-lactams (cefepime, ceftolozane-tazobactam, and ceftobiprole) and other non-beta-lactam antibiotics (tobramycin). Therapeutic drug monitoring for aminoglycosides was performed according to the treating physician’s request, either before or during the OPAT program. Additionally, oral sequential therapy, hospital stay duration, treatment duration (including in-hospital treatment, OPAT treatment and sequential oral therapy), clinical outcomes (including unplanned readmission, infection progression, adverse events (AEs), or death during OPAT and 30 days after treatment end), and safety outcomes (AEs and vascular access complications) were collected.

The primary outcomes were treatment failure during the OPAT, defined as unplanned readmission (for any cause, regardless of relation to the prior infection), infection progression, adverse events, or death occurring during the OPAT episode; and 30-day treatment failure, a composite endpoint defined as unplanned readmission for any cause, infection relapse without hospitalization (defined as new positive cultures for *P. aeruginosa*), or death from any cause within 30 days following completion of OPAT. In cases of 30-day treatment failure, microbiological data were specifically reviewed to evaluate the emergence of antimicrobial resistance during therapy. Secondary outcomes included safety outcomes, assessed through monitoring of adverse events and catheter-related complications.

### 2.4. Statistical Analysis

An initial descriptive analysis was carried out to summarize the baseline characteristics of the study population and the primary and secondary outcomes. Categorical variables were expressed as percentages, while continuous variables were presented as median values with interquartile ranges (IQR). Comparisons of continuous variables were performed using the Mann–Whitney U test, whereas categorical variables were compared using either the chi-square test or Fisher’s exact test, as appropriate.

To identify factors associated with treatment failure during the OPAT episode and within 30 days’ post-treatment, both bivariate and multivariable analyses were carried out. Bivariate analysis was first performed to explore potential associations. Variables with a *p*-value < 0.2 in the bivariate analysis were further evaluated using logistic regression, and those with a *p*-value < 0.05 were considered candidates for inclusion in the multivariable logistic regression model. Multivariable analysis was then performed to estimate odds ratios (ORs) and corresponding 95% confidence intervals (CIs). Variables included in the multivariable model were also those considered clinically relevant or previously reported to be associated with poor outcomes. No data were missing for variables included in the multivariable model.

All statistical analyses were performed using SPSS software version 28.0 (SPSS Inc., Chicago, IL, USA).

## 3. Results

### 3.1. Population and Treatment Characteristics

Between November 2012 and December 2024, a total of 391 patients with *P. aeruginosa* infection were enrolled in the DOMUS OPAT programme. After excluding 98 patients with polymicrobial infections and 3 receiving palliative end-of-life care, 290 patients were included in the analysis. Their baseline characteristics are summarized in Table 1. Over the 13-year study period (2012–2024), the annual number of OPAT patients with *P. aeruginosa* infection showed a progressive increasing trend, with the highest number of cases observed in 2024 (*n* = 30) (Figure S1).

The most common diagnoses were bronchiectasis exacerbation (*n* = 115, 39.7%) and complicated urinary tract infections (*n* = 44, 15.2%), followed by pneumonia (*n* = 24, 8.6%), skin and soft tissue infections (*n* = 24, 8.3%), exacerbated COPD (*n* = 18, 6.2%), osteoarticular infections (*n* = 16, 5.5%) and endovascular infections (*n* = 11, 3.8%). Endovascular infections constituted a heterogeneous group, including infective endocarditis, vascular prosthesis infections, and catheter-related bloodstream infections. Source control was considered necessary in 92 patients. Before inclusion in the OPAT programme, source control had been performed in 71 cases (77.2%), while 21 patients (22.8%) initiated OPAT without prior source control.

Regarding resistance patterns, 1.7% (*n* = 5) of isolates were classified as DTR. Two patients were treated with ceftazidime-avibactam plus amikacin, while the remaining three received ceftolozane-tazobactam, ceftobiprole, and tobramycin, respectively.

A total of 72.8% (*n* = 211) of patients received monotherapy. The most commonly used antimicrobials for monotherapy were ceftazidime (*n* = 130, 44.8%), piperacillin-tazobactam (*n* = 50, 17.2%), and meropenem (*n* = 18, 6.2%). Combination therapy was administered to 27.2% (*n* = 79) of patients, always involving a beta-lactam and an aminoglycoside, and was predominantly used for bronchiectasis exacerbations (79.7%, *n* = 63). Most of these patients had bronchiectasis 79.7% (*n* = 63), followed by pneumonia 10.1% (*n* = 8), exacerbated COPD 7.6% (*n* = 6) and endovascular infection 2.5% (*n* = 2). Baseline characteristics of patients according to antibiotic treatment strategy (combination therapy vs. monotherapy) are shown in Table S1.

As shown in Table 2, the median length of hospital stay prior to OPAT was 5.5 days (range (0–10)), with 105 patients (36.2%) receiving OPAT without prior hospitalization. The median duration of inpatient intravenous antimicrobials therapy was 3 days (IQR 0–7), the median duration of OPAT was 10 days (IQR 7–14) with a median total treatment duration of 14 days (IQR 12–19).

### 3.2. Clinical Outcomes

The main clinical outcomes are summarized in Table 2. The rate of treatment failure during OPAT was 7.6% (*n* = 22). In the 30 days following OPAT, 45 patients (15.5%) experienced treatment failure. Overall, 77% of patients (*n* = 223/290) achieved clinical success.

During OPAT, the main causes of treatment failure were unplanned readmission in 72.7% (*n* = 16) (13 were related to the infection and 3 were unrelated), infection progression (not requiered hospitalization) in 13.6% (*n* = 3), and AEs, which are described later, in 13.6% (*n* = 3). The 3 unrelated readmissions corresponded to urgent procedures not associated with the infection (one case of acute cholecystitis and two trauma-related events), and no patients died during OPAT.

Within 30 days following the end of OPAT, unplanned readmissions, all related to the prior infection, occurred in 66.6% (*n* = 30), infection relapse (not requiered hospitalization) in 17.8% (*n* = 8), and death in 15.6% (*n* = 7). Of the 7 deaths, 6 were related to the infection, while one was not. This latter patient had been included in the DOMUS program for bronchiectasis exacerbation, but death was due to hepatic encephalopathy as the immediate cause, in the context of alcohol-related cirrhosis. Among the six infection-related deaths, the underlying infections were bronchiectasis (33.3%, *n* = 2), intra-abdominal or anorectal infection/abscess (16.7%, *n* = 1), pneumonia (16.7%, *n* = 1), complicated urinary tract infection (16.7%, *n* = 1), and skin and soft tissue infection (16.7%, *n* = 1).

Microbiological data from patients with 30-day treatment failure are detailed in Table S2. At baseline, 57.8% (*n* = 26) of the isolated strains were susceptible to the administered regimen, while 20% (*n* = 9) showed intermediate susceptibility; in 22.2% (*n* = 10), the antibiogram was not available due to a change in the hospital information system. In patients with available baseline susceptibility data (*n* = 35), post-treatment antibiogram results were analyzed. In 57.1% (*n* = 20) of cases, *P. aeruginosa* did not regrow, whereas in the remaining cases (*n* = 15), the emergence of resistance was documented in only 13.3% (*n* = 6), corresponding to a transition from susceptible to resistant. Notably, none of the patients with intermediate baseline susceptibility (*n* = 9) developed resistance.

Of the 6 patients who developed resistance, 4 had been treated with ceftazidime and 2 with piperacillin–tazobactam. Clinical outcomes in this subgroup included one death attributable to the infection.

Vascular access complications were observed in 32.6% (*n* = 95) of patients, mainly due to extravasation, malfunction, or catheter loss (68.4%, *n* = 65) and phlebitis (31.6%, *n* = 30). These complications were more frequent in patients with peripheral catheters (81.05%, *n* = 77). AEs were reported in 3.2% (*n* = 3) of patients: skin reactions occurred in two patients receiving ceftazidime, and dizziness and gastrointestinal disorders occurred in one patient associated with piperacillin-tazobactam.

The need for source control and its potential association with treatment outcomes was also explored. No statistically significant association was observed either during OPAT or within 30 days after OPAT completion. In addition, no significant differences in source control rates were observed among infections in which source control was feasible.

### 3.3. Bivariate and Multivariable Analysis Model

The results of the bivariate and multivariable analysis are shown in Table 3, Table 4 and Table 5 and in Supplementary Tables S3 and S4.

#### 3.3.1. Treatment Failure During OPAT

In the bivariate analysis (Table 3), variables associated with OPAT failure at a *p*-value < 0.2 included male sex (*p* = 0.015), COPD comorbidity (*p* = 0.116), endovascular infection (*p* = 0.199), treatment with meropenem (*p* = 0.146), and with other β-lactams (cefepime, ceftolozane-tazobactam, and ceftobiprole) (*p* = 0.171). These variables were subsequently included in the logistic regression analysis to assess their independent effect on the risk of treatment failure (Table S3). Variables included in the multivariable model, considering both statistical significance and clinical relevance are shown in Table 5. Sex (male) and COPD comorbidity were independently associated with treatment failure during OPAT, with increases in risk exceeding fourfold and threefold, respectively.

#### 3.3.2. Treatment Failure 30 Days After OPAT Treatment End

In the bivariate analysis (Table 4), variables associated with 30-day treatment failure at a *p*-value < 0.2 included age (*p* = 0.032), male sex (*p* = 0.086), Charlson comorbidity index (*p*< 0.001), COPD comorbidity (*p* = 0.138), chronic heart failure (*p* = 0.011), malignancy (*p* = 0.002), intra-abdominal or anorectal infection (*p* = 0.010), exacerbated COPD (*p* = 0.011), bronchiectasis exacerbation (*p* = 0.108), lung abscess (*p* = 0.078), endovascular infection (*p* = 0.156), osteoarticular infection (*p* = 0.145), other β-lactams (cefepime, ceftolozane-tazobactam, and ceftobiprole) (*p* = 0.191), and other non-β-lactam antimicrobials (tobramycin) (*p* = 0.064). By the contrary, combination therapy with β-lactams plus aminoglycosides was associated with a lower risk of 30-day treatment failure (*p* = 0.008). These variables were subsequently included in the logistic regression analysis to assess their independent effect on the risk of treatment failure (Table S4). Variables included in the multivariable model, based on both statistical and clinical relevance are shown in Table 5. Charlson score, COPD exacerbation, and endovascular infection were independently associated with 30-day treatment failure. However, combination therapy was associated with a threefold lower risk of failure compared to monotherapy. The variable other non-beta-lactam antimicrobials was not included in the multivariable analysis due to the limited number of cases (*n* = 3).

## 4. Discussion

OPAT has been proposed as a strategy to manage *P. aeruginosa* infections in clinically stable patients, although the experience supporting this approach remains limited [9,10,11,12]. In our cohort of 290 patients, the most frequent infections were bronchiectasis exacerbations and complicated urinary tract infections. Most patients received monotherapy, primarily with ceftazidime, while 27% received combination therapy with a beta-lactam and an aminoglycoside. Vascular access complications were relatively common, while antimicrobials-related AEs were infrequent. Treatment failure occurred in 22 patients (7.6%) during OPAT, and in 45 patients (15.5%) within 30 days, with an overall clinical success rate of 77% (*n* = 223/290). Factors associated with failure during OPAT and 30-day failure were identified.

The results of our study are consistent with the available literature, although the evidence remains scarce and heterogeneous. Several studies and case series have focused exclusively on *P. aeruginosa*, predominantly addressing respiratory infections, osteomyelitis, and complicated soft tissue infections [11,15,16,17]. Among them, the majority used continuous infusion [11,17] and/or elastomeric pumps [15,16] to deliver antipseudomonal β-lactams such as piperacillin/tazobactam and ceftolozane/tazobactam. These studies reported high clinical effectiveness at the end of treatment, generally above 80–90%. AEs were not reported, and vascular access complications occurred in 8–29% of cases. These results are comparable to those observed in our study in terms of both effectiveness and safety, demonstrating that OPAT is feasible and well tolerated in selected patients. In larger real-world cohorts [18,19,20] including infections caused by different microorganisms, *P. aeruginosa* was isolated in around 38–41% of cases, predominantly in respiratory infections. In those studies, piperacillin/tazobactam was the most frequently used antimicrobial. In our cohort, piperacillin/tazobactam was the second most frequently used antimicrobial after ceftazidime. This preference for ceftazidime likely reflects its narrower spectrum against anaerobes and lower impact on colonic microbiota. Across these studies, OPAT was generally safe and well tolerated with 12–20% AEs, including catheter-related, and cure or improvement rates ranging from 84% to 93%. However, 30-day mortality in some studies reached 24.7% [18], and 90-day OPAT-related hospital readmission was 13% [20], markedly higher than in our cohort, where 30-day mortality was 2.4%. This is likely because we excluded terminally ill patients, who represented nearly half of deaths in the previous studies [18]. While these findings provide useful context regarding the effectiveness and safety of OPAT in real-world settings, they are not directly comparable to our results, as these cohorts included a broader spectrum of microorganisms. Finally, individual case reports have shown successful outpatient management of complex *P. aeruginosa* infections such as osteomyelitis with prolonged courses of ceftolozane/tazobactam, ceftazidime, or cefiderocol, achieving clinical cure without major safety concerns [10,13,21]. Overall, our findings complement and expand the current literature, supporting OPAT as a feasible and safe approach for the outpatient treatment of *P. aeruginosa* infections.

In our cohort, male sex and COPD comorbidity were associated with a higher risk of therapeutic failure during OPAT. To our knowledge, this is the first study analysing which factors are associated with therapeutic failure during OPAT in patients with *P. aeruginosa* infections. Previous studies, by contrast, have focused only on 30-day outcomes. Male sex and COPD comorbidity were associated with failure during OPAT. COPD patients may represent a population in whom infections tend to become chronic and difficult to treat, or resistant [22]. Regarding male sex, although multivariable analysis identified it as an independent predictor, it did not capture other factors that may contribute to the higher failure rate observed in men. Male patients in our cohort had a higher comorbidity burden, with 77% (41/53) having diabetes, 71% (63/89) heart failure, 71% (60/85) malignancy, and 95% (21/22) renal failure, and they more frequently presented with severe infections, including pulmonary abscesses 86% (6/7), endovascular infections 82% (14/17), and osteoarticular infections 81% (13/16). The median Charlson comorbidity index was also higher in men (2 vs. 1 in women). Taken together, these findings suggest that the higher risk of failure observed in men may not be attributable solely to biological sex, but rather to greater comorbidity and more severe clinical presentations. This highlights the need for careful risk stratification before initiating OPAT, taking into account both demographic characteristics and overall comorbidity burden. Regarding meropenem, treatment with this antibiotic was not statiscally associated with poorer evolution, although a trend toward worse outcomes was observed. Only 18 patients in our cohort received meropenem, all presenting with highly heterogeneous and particularly complex infections. Treatment failure occurred in three patients during OPAT and in four ones within 30 days. Notably, two of these cases involved endovascular infections, one of which required adjunctive phage therapy, and in two others, complete source control was not achieved. Given the small number of patients and the high clinical complexity, drawing definitive conclusions about meropenem’s effectiveness in this setting is difficult, particularly because meropenem was mainly reserved for the most severe cases or those with prior treatment failure, which may reflect underlying disease severity rather than the drug’s efficacy.

Regarding 30-day failure, we observed significant associations with a higher Charlson comorbidity index, COPD exacerbations, and endovascular infections. The relevance of comorbidity burden is supported by recent data from Ferro Rodríguez et al. [18], who reported poorer outcomes in patients with malignancy and higher mortality in those with heart failure, both variables included in the Charlson index. In addition, Durojaiye et al. [23] developed a prediction model for unplanned 30-day hospitalization among OPAT patients, identifying comorbidity burden and endovascular infection as key predictors. Nevertheless, the clinical significance of this finding should be interpreted cautiously, as only 11 patients had endovascular infections. Similarly, the number of patients with COPD exacerbations was small (*n* = 18); however, acute exacerbations are known to negatively influence COPD progression and increase relapse risk [24]. All cases of 30-day failure in this subgroup (*n* = 7) occurred in men, who also tended to have a higher comorbidity burden, although no significant association with sex was demonstrated. Combination therapy with beta-lactams plus aminoglycosides was independently associated with a lower risk of 30-day failure. However, this finding contrasts with evidence from randomized controlled trials and recent meta-analyses [25]. A plausible explanation is confounding by indication: among the 79 patients receiving combination therapy in our cohort, 57% were women and 79.7% had bronchiectasis (Table S1), suggesting that this regimen may have been preferentially used in patients with chronic respiratory conditions or exacerbations, who generally have a better short-term prognosis than those with more severe invasive infections [26]. Although these characteristics were not identified as independent predictors in multivariable analysis, they may partly account for the observed protective association, rather than reflecting a true therapeutic advantage of combination regimens. Therefore, this association should be interpreted with caution, as it likely reflects residual confounding rather than a true therapeutic benefit.

This study has several limitations. First, its observational design carries an inherent risk of bias due to unmeasured confounders, including clinical discretion in patient selection. Second, despite the large overall sample size, some subgroups, such as endovascular infections, were too small to support definitive conclusions. Third, heterogeneity in diagnoses, antimicrobial regimens, and treatment durations may limit the applicability of results to more homogeneous populations. In addition, post-treatment microbiological follow-up was not systematically performed, potentially underestimating recurrent infection. Furthermore, the absence of molecular typing precluded the distinction between relapse and reinfection in patients with multiple episodes, although the number of such cases was limited. Nevertheless, the study’s strengths include its prospective nature, the large real-world cohort, and the highly structured OPAT model with close nursing and specialist monitoring. These elements enhance the robustness of the findings and support the feasibility of OPAT for complex *P. aeruginosa* infections.

## 5. Conclusions

In conclusion, our results support OPAT as an effective and safe strategy for managing *P. aeruginosa* infections in clinically stable patients. Patients with COPD, either as a comorbidity or during an exacerbation, and those with a higher Charlson score may require closer follow-up. Overall, these findings underscore the value of well-structured OPAT programs in the management of complex *P. aeruginosa infections* although further studies are needed to confirm these results. Importantly, in selected high-risk patients, OPAT may be initiated despite a recognized likelihood of readmission, prioritizing patient comfort and time spent at home, tailored to each patient’s individual situation.

## Figures and Tables

**Table 1:** Baseline and infection characteristics.

	All Cases (*n* = 290)
**Baseline Characteristics**	
Median age (IQR)	68 (57–79)
Gender, male	166 (57.2)
Comorbidities	
Median Charlson score (IQR)	2 (1–3)
Diabetes mellitus	53 (18.3)
COPD comorbidity	151 (52.1)
Chronic renal failure	22 (7.6)
Chronic heart failure	89 (30.7)
Malignancy	85 (29.3)
Chronic liver disease	14 (4.8)
**Related to infection**	
Diagnosis	
Intra-abdominal or anorectal infection or abscess	12 (4.1)
Pneumonia	25 (8.6)
Exacerbated COPD	18 (6.2)
Bronchiectasis exacerbation	115 (39.7)
Lung abscess	7 (2.4)
Complicated Urinary Tract Infection	44 (15.2)
Skin and soft tissue infection	24 (8.3)
Endovascular infection	11 (3.8)
Osteoarticular infection	16 (5.5)
Others	18 (6.2)
Control of the source of infection	
Yes	71 (24.5)
No	21 (7.2)
Not applicable	198 (68.3)

Data are presented as *n* (%) unless indicated otherwise. Abbreviation: IQR—interquartile range; COPD—chronic obstructive pulmonary disease.

**Table 2:** Treatment characteristics and clinical outcomes.

	All Cases (*n* = 290)
**Treatment Characteristics**	
Antimicrobial treatment	
Combination therapy (beta-lactams + aminoglycosides)	79 (27.2)
Monotherapy	211 (72.8)
Ceftazidime	130 (44.8)
Piperacillin-tazobactam	50 (17.2)
Meropenem	18 (6.2)
Other beta-lactams *	10 (3.4)
Other non-beta-lactam antimicrobial **	3 (1)
Sequential therapy with quinolones	13 (4.5)
Length of hospital stay in days, median (IQR)	5.5 (0–10)
Inpatient treatment days, median (IQR)	3 (0–7)
OPAT treatment days, median (IQR)	10 (7–14)
Total treatment duration days, median (IQR)	14 (12–19)
**Clinical outcomes**	
Effectiveness	
During OPAT time	
Treatment failure	22 (7.6)
Unplanned readmission	16 (5.5)
Infection progression	3 (1)
Adverse events	3 (1)
Death during OPAT	0
30-day Treatment failure (composite endpoint)	45 (15.5)
30-day readmission (related to prior infection)	30 (10.3)
Infection relapse	8 (2.8)
Death	7 (2.4)
Safety	
Vascular access complication	95 (32.8)
Type of complication	
Phlebitis	30 (10.3)
Extravasation, malfunction or catheter loss	65 (22.4)
Type of catheter	
Peripheral	77 (81.05)
Midline	16 (16.84)
Others	2 (2.11)
Adverse events	3 (1)

Data are presented as *n* (%) unless indicated otherwise. Abbreviation: IQR—interquartile range; OPAT—Outpatient parenteral antimicrobial treatment. * Cefepime (*n* = 5), ceftolozane-tazobactam (*n* = 4) and ceftobiprole (*n* = 1) ** Tobramycin (*n* = 3).

**Table 3:** Bivariate analysis of variables associated with treatment failure during OPAT treatment.

			Treatment Success During OPAT Time (*n* = 268)	Treatment Failure During OPAT Time (*n* = 22)	*p* Value ^a^
**Baseline Characteristics**					
Median age (IQR)			67.5 (57–78.8)	69 (54.3–79)	0.789 ^b^
Gender, male			148 (55.2)	18 (81.8)	**0.015 ^a^**
Comorbidities					
	Median Charlson score (IQR)		2 (1–3)	2 (1–4)	0.390 ^b^
	Diabetes mellitus		47 (17.5)	6 (27.3)	0.256 ^c^
	COPD comorbidity		136 (50.7)	15 (68.2)	**0.116 ^a^**
	Chronic renal failure		19 (7.1)	3 (13.6)	0.226 ^c^
	Chronic heart failure		82 (30.6)	7 (31.8)	0.905 ^a^
	Malignancy		79 (29.5)	6 (27.3)	0.827 ^a^
	Chronic liver disease		14 (5.2)	0	0.610 ^c^
**Related to infection**					
Diagnosis					
	Intra-abdominal or anorectal infection or abscess		11 (4.1)	1 (4.5)	1.000 ^c^
	Pneumonia		23 (8.6)	2 (9.1)	1.000 ^c^
	Exacerbated COPD		16 (6)	2 (9.1)	0.636 ^c^
	Bronchiectasis exacerbation		105 (39.2)	10 (45.5)	0.563 ^a^
	Lung abscess		6 (2.2)	1 (4.5)	0.428 ^c^
	Complicated Urinary Tract Infection		43 (16)	1 (4.5)	0.218 ^c^
	Skin and soft tissue infection		26 (8.6)	1 (4.5)	1.000 ^c^
	Endovascular infection		9 (3.4)	2 (9.1)	**0.199 ^c^**
	Osteoarticular infection		14 (5.2)	2 (9.1)	0.347 ^c^
	Others		18 (6.7)	0	0.377 ^c^
Control of source of infection					
	Yes (*n* = 71)		66 (92.9)	5 (7)	0.657 ^c^
	Not (*n* = 21)		19 (90.4)	2 (9.5)	
**Treatment Characteristics**					
Antimicrobial treatment					
	Combination therapy (beta-lactams + aminoglycosides)		74 (27.6)	5 (22.7)	0.621 ^a^
	Monotherapy		194 (72.4)	17 (77.3)	
		Ceftazidime	21 (45.1)	9 (40.9)	0.726 ^a^
		Piperacillin-tazobactam	48 (17.9)	2 (9.1)	0.389 ^c^
		Meropenem	15 (5.6)	3 (13.6)	**0.146 ^c^**
		Other beta-lactams	8 (3)	2 (9.1)	**0.171 ^c^**
		Other non-beta-lactam antimicrobial	2 (0.7)	1 (4.5)	0.211 ^c^
Total treatment duration days, median (IQR)			14 (12–19)	13.5 (8–19.5)	0.270 ^b^

Data are presented as *n* (%) unless indicated otherwise. Abbreviation: IQR—interquartile range; OPAT—Outpatient parenteral antimicrobial treatment; ^a^ P values were calculated by chi-square test, except where otherwise specified. ^b^ Mann–Whitney U-test. ^c^ Fisher’s test.

**Table 4:** Bivariate analysis of variables associated with 30-day treatment failure.

			30 Day Treatment Success (*n* = 245)	30 Day Treatment Failure (*n* = 45)	*p* Value ^a^
**Baseline Characteristics**					
Median age (IQR)			67 (54–78)	75 (60–80)	**0.032 ^b^**
Gender, male			135 (55.1)	31 (68.9)	**0.086 ^a^**
Comorbidities					
	Median Charlson score (IQR)		2 (1–3)	3 (2–4)	**0.000 ^b^**
	Diabetes mellitus		42 (17.1)	11 (24.4)	0.244 ^a^
	COPD comorbidity		123 (50.2)	28 (62.2)	**0.138 ^a^**
	Chronic renal failure		19 (7.8)	3 (6.7)	1.000 ^c^
	Chronic heart failure		68 (27.8)	21 (46.7)	**0.011 ^a^**
	Malignancy		63 (25.7)	22 (48.9)	**0.002 ^a^**
	Chronic liver disease		11 (4.5)	3 (6.7)	0.462 ^c^
**Related to infection**					
Diagnosis					
	Intra-abdominal or anorectal infection or abscess		7 (2.9)	5 (11.1)	**0.025 ^c^**
	Pneumonia		22 (9)	3 (6.7)	0.777 ^c^
	Exacerbated COPD		11 (4.5)	7 (15.6)	**0.011 ^c^**
	Bronchiectasis exacerbation		102 (41.6)	13 (28.9)	**0.108 ^a^**
	Lung abscess		4 (1.6)	3 (6.7)	**0.078 ^c^**
	Complicated Urinary Tract Infection		40 (16.3)	4 (8.9)	0.201 ^a^
	Skin and soft tissue infection		21 (8.6)	3 (6.7)	1.000 ^c^
	Endovascular infection		6 (2.4)	5 (11.1)	**0.016 ^c^**
	Osteoarticular infection		16 (6.5)	0	**0.145 ^c^**
	Others		16 (6.5)	2 (4.4)	1.000 ^c^
Control of source of infection					
	Yes (*n* = 71)		58 (81.7)	13 (18.3)	0.226 ^c^
	Not (*n* = 21)		14 (66.7)	7 (33.3)	
**Treatment Characteristics**					
Antimicrobial treatment					
	Combination therapy (beta-lactams + aminoglycosides)		74 (30.2)	5 (11.1)	**0.008 ^a^**
	Monotherapy				
		Ceftazidime	108 (44.5)	22 (46.7)	0.551 ^a^
		Piperacillin-tazobactam	41 (16.7)	9 (20)	0.594 ^a^
		Meropenem	14 (5.7)	4 (8.9)	0.497 ^c^
		Other beta-lactams	7 (2.9)	3 (6.7)	**0.191 ^c^**
		Other non-beta-lactam antimicrobial	1 (0.4)	2 (4.4)	**0.064 ^c^**
Total treatment duration days, median (IQR)			14 (12–18)	14 (11.5–21)	0.508 ^b^

Data are presented as *n* (%) unless indicated otherwise. Abbreviation: IQR—interquartile range; OPAT—Outpatient parenteral antimicrobial treatment. ^a^ P values were calculated by chi-square test, except where otherwise specified. ^b^ Mann–Whitney U-test. ^c^ Fisher’s test.

**Table 5:** Multivariable logistic regression analysis of variables associated with treatment failure during OPAT and with 30-day treatment failure.

	*p* Value	OR (CI95%)
**Failure during OPAT**		
Gender, male	**0.012**	4.239 (1.365–13.168)
COPD comorbidity	**0.041**	3.075 (1.048–9.025)
Endovascular infection	0.123	4.312 (0.674–27.589)
Meropenem	0.158	2.799 (0.672–11.661)
Other beta-lactams	0.224	2.914 (0.520–16.335)
**30-day treatment failure**		
Age	0.085	1.022 (0.997–1.049)
Charlson score	**0.007**	1.262 (1.064–1.496)
Intra-abdominal or anorectal infection or abscess	0.070	3.322 (0.905–12.201)
Exacerbated COPD	**0.007**	4.452 (1.502–13.195)
Endovascular infection	**0.005**	6.790 (1.785–25.827)
Combination therapy (beta-lactams + aminoglycosides)	**0.040**	0.343 (0.123–0.954)

OR: odds ratio; CI: confidence interval.

## Data Availability

All data relevant to the study are included in the article or uploaded as Supplementary Information.

## Supplementary Materials

The following supporting information can be downloaded at: https://www.mdpi.com/article/10.3390/pharmaceutics18050549/s1. Table S1. Baseline characteristics of patients according to antibiotic treatment strategy (combination therapy vs. monotherapy); Tabla S2. Microbiological evolution in *Pseudomonas aeruginosa* among patients with 30-day treatment failure; Table S3. Logistic regression analysis of factors associated with treatment failure during OPAT; Table S4. Logistic regression analysis of factors associated with 30-day treatment failure; Figure S1. Annual number of OPAT patients with *Pseudomonas aeruginosa* infection from 2012 to 2024.

## References

[1] Reidy P., Breslin T., Muldoon E. (2024). Outpatient parenteral antimicrobial therapy (OPAT) across the world: A comparative analysis-what lessons can we learn?. JAC Antimicrob. Resist..

[2] Norris A.H., Shrestha N.K., Allison G.M., Keller S.C., Bhavan K.P., Zurlo J.J., Hersh A.L., Gorski L.A., Bosso J.A., Rathore M.H. (2019). 2018 Infectious Diseases Society of America Clinical Practice Guideline for the Management of Outpatient Parenteral Antimicrobial Therapy. Clin. Infect. Dis..

[3] Staples J.A., Ho M., Ferris D., Hayek J., Liu G., Tran K.C., Sutherland J.M. (2022). Outpatient Versus Inpatient Intravenous Antimicrobial Therapy: A Population-Based Observational Cohort Study of Adverse Events and Costs. Clin. Infect. Dis..

[4] Mohammed S.A., Cotta M.O., Assefa G.M., Erku D., Sime F. (2024). Barriers and facilitators for the implementation and expansion of outpatient parenteral antimicrobial therapy: A systematic review. J. Hosp. Infect..

[5] Schwartz B., Klamer K., Zimmerman J., Kale-Pradhan P.B., Bhargava A. (2024). Multidrug Resistant *Pseudomonas aeruginosa* in Clinical Settings: A Review of Resistance Mechanisms and Treatment Strategies. Pathogens.

[6] Papadimitriou-Olivgeris M., Jacot D., Guery B. (2022). How to Manage *Pseudomonas aeruginosa* Infections. Adv. Exp. Med. Biol..

[7] EUCAST (2021). To Clinical Colleagues on Recent Changes in Clinical Microbiology Susceptibility Reports.

[8] Tamma P.D., Aitken S.L., Bonomo R.A., Mathers A.J., van Duin D., Clancy C.J. (2023). Infectious Diseases Society of America 2023 Guidance on the Treatment of Antimicrobial Resistant Gram-Negative Infections. Clin. Infect. Dis..

[9] Cosentino F., Viale P., Giannella M. (2023). MDR/XDR/PDR or DTR? Which definition best fits the resistance profile of *Pseudomonas aeruginosa*?. Curr. Opin. Infect. Dis..

[10] Conde-Díaz C., Llenas-García J., Parra Grande M., Terol Esclapez G., Masiá M., Gutiérrez F. (2017). Severe skull base osteomyelitis caused by *Pseudomonas aeruginosa* with successful outcome after prolonged outpatient therapy with continuous infusion of ceftazidime and oral ciprofloxacin: A case report. J. Med. Case Rep..

[11] Venuti F., Gaviraghi A., De Nicolò A., Stroffolini G., Longo B.M., Di Vincenzo A., Ranzani F.A., Quaranta M., Romano F., Catellani E. (2023). Real-Life Experience of Continuously Infused Ceftolozane/Tazobactam in Patients with Bronchiectasis and Multidrug-Resistant *Pseudomonas aeruginosa* Infection in the Outpatient Setting. Antibiotics.

[12] Goncette V., Layios N., Descy J., Frippiat F. (2021). Continuous infusion, therapeutic drug monitoring and outpatient parenteral antimicrobial therapy with ceftazidime/avibactam: A retrospective cohort study. J. Glob. Antimicrob. Resist..

[13] Álvarez Otero J., Lamas Ferreiro J.L., Sanjurjo Rivo A., de la Fuente Aguado J. (2020). Outpatient Parenteral Antimicrobial Therapy With Ceftolozane/Tazobactam via Continuous Infusion for Multidrug-Resistant *Pseudomonas aeruginosa* Osteomyelitis. Open Forum Infect. Dis..

[14] Von Elm E., Altman D.G., Egger M., Pocock S.J., Gøtzsche P.C., Vandenbroucke J.P. (2007). The Strengthening the Reporting of Observational Studies in Epidemiology (STROBE) statement: Guidelines for reporting observational studies. Bull. World Health Organ..

[15] Ferreiro J.L.L., Otero J.Á., Rivo A.S., González L.G., Conde I.R., Soneira M.F., García J.P., de la Fuente Aguado J. (2021). Outpatient therapy with piperacillin/tazobactam using elastomeric pumps in patients with *Pseudomonas aeruginosa* infection. Sci. Rep..

[16] Giuliano G., Tarantino D., Tamburrini E., Nurchis M.C., Scoppettuolo G., Raffaelli F. (2024). Effectiveness and safety of Ceftolozane/Tazobactam administered in continuous infusion through elastomeric pumps in OPAT regimen: A case series. Infect. Dis..

[17] Jones B.M., Huelfer K., Bland C.M. (2020). Clinical and Safety Evaluation of Continuously Infused Ceftolozane/Tazobactam in the Outpatient Setting. Open Forum Infect. Dis..

[18] Ferro Rodríguez S., Chantres Legaspi Y., Romay Lema E.M., Ayuso García B., Castellano Copa P., Peinó Camba P., Barcia Losada A., Rodríguez Díaz C. (2024). [Translated article] Retrospective study of home antibiotic infusion therapy using elastomeric infusion pumps. Farm. Hosp..

[19] Ponce González M.A., Mirón Rubio M., Mujal Martinez A., Estrada Cuxart O., Fiuza Perez D., Salas Reinoso L., Fernandez Fabrellas E., Chiner Vives E. (2017). Effectiveness and safety of outpatient parenteral antimicrobial therapy in acute exacerbation of chronic obstructive pulmonary disease. Int. J. Clin. Pract..

[20] Forier B., Schaevers V., Spriet I., Quintens C., Desmet S., Bos S., Van Bleyenbergh P., Lorent N., De Sadeleer L., Godinas L. (2025). Outpatient parenteral antibiotic therapy in non-cystic fibrosis lung transplant recipients: Characteristics, efficacy and safety. Eur. J. Clin. Microbiol. Infect. Dis..

[21] García-Queiruga M., Feal Cortizas B., Lamelo Alfonsín F., Pertega Diaz S., Martín-Herranz I. (2021). Continuous infusion of antibiotics using elastomeric pumps in the hospital at home setting. Rev. Esp. Quimioter..

[22] Schellong P., Wennek-Klose J., Spiegel C., Rödel J., Hagel S. (2024). Successful outpatient parenteral antibiotic therapy with cefiderocol for osteomyelitis caused by multi-drug resistant Gram-negative bacteria: A case report. JAC Antimicrob. Resist..

[23] Durojaiye O.C., Kritsotakis E.I., Johnston P., Kenny T., Ntziora F., Cartwright K. (2019). Developing a risk prediction model for 30-day unplanned hospitalization in patients receiving outpatient parenteral antimicrobial therapy. Clin. Microbiol. Infect..

[24] MacLeod M., Papi A., Contoli M., Beghé B., Celli B.R., Wedzicha J.A., Fabbri L.M. (2021). Chronic obstructive pulmonary disease exacerbation fundamentals: Diagnosis, treatment, prevention and disease impact. Respirology.

[25] Tekes-Manuva D., Babich T., Kozlovski D., Elbaz M., Yahav D., Halperin E., Leibovici L., Avni T. (2025). What is the most effective antibiotic monotherapy for severe *Pseudomonas aeruginosa* infection? A systematic review and meta-analysis of randomized controlled trials. Clin. Microbiol. Infect..

[26] López-Cortés L.E., Ayerbe-García R., Carrasco-Hernández L., Fraile-Ramos E., Carmona-Caballero J.M., Quintana-Gallego E., Valido-Morales A., Praena J., Pachón-Diaz J., DOMUS OPAT Group (2019). Outpatient Parenteral Antimicrobial Treatment for Non-Cystic Fibrosis Bronchiectasis Exacerbations: A Prospective Multicentre Observational Cohort Study. Respiration.

