# Supplementary material for: Outpatient Parenteral Antimicrobial Therapy for Pseudomonas aeruginosa Infections: Effectiveness and Safety
Source: Pharmaceutics. 2026 Apr 29;18(5):549. doi: 10.3390/pharmaceutics18050549 (PMC13210217; doi:10.3390/pharmaceutics18050549)

Table S1. Baseline characteristics of patients according to antibiotic treatment strategy (combination therapy vs monotherapy)

	Combination therapy (beta-lactams + aminoglycosides) 79 (27.2)	Monotherapy 211 (72.8)	*p* value[a]
**Baseline Characteristics**			
Median age (IQR)	66 (49-79)	68 (57-79)	0.460[b]
Gender, male	34 (43)	132 (62.6)	0.003[a]
Comorbidities			
Median Charlson score (IQR)	1 (1-3)	2 (1-4)	0.016[b]
Diabetes mellitus	8 (10.1)	45 (21.3)	0.028[a]
COPD comorbidity	66 (83.5)	85 (40.3)	0.000[a]
Chronic renal failure	2 (2.5)	20 (9.5)	0.047[a]
Chronic heart failure	18 (22.8)	71 (33.6)	0.074[a]
Malignancy	15 (19)	70 (33.2)	0.018[a]
Chronic liver disease	2 (2.5)	12 (5.7)	0.364[c]
**Related to infection**			
Diagnosis			
Intra-abdominal or anorectal infection or abscess	0	12 (5.7)	0.041[c]
Pneumonia	8 (10.1)	17 (8.1)	0.576[a]
Exacerbated COPD	6 (7.6)	12 (5.7)	0.587[c]
Bronchiectasis exacerbation	63 (79.7)	52 (24.6)	0.000[a]
Lung abscess	0	7 (3.3)	0.196[c]
Complicated Urinary Tract Infection	0	44 (20.9)	0.000[a]
Skin and soft tissue infection	0	24 (11.4)	0.002[a]
Endovascular infection	2 (2.5)	9 (4.3)	0.733[c]
Osteoarticular infection	0	16 (7.6)	0.008[c]
Others	0	18 (8.5)	0.005[c]

[a] P values were calculated by chi-square test, except where otherwise specified. [b] Mann-Whitney U-test
[c]Fisher test

Tabla S2. Microbiological evolution in *Pseudomonas aeruginosa* among patients with 30-day treatment failure.

**Patients with 30-day treatment failure**	**N=45**
**Baseline susceptibility to administered regimen (N=45)**	
Susceptible	26 (57,8%)
Intermediate	9 (20%)
Not available	10 (22.2%)
**Antibiogram results after antibiotic treatment (N=35)**	
Isolates in which *P. aeruginosa* does not regrow	20 (57.1%)
Isolates in which *P. aeruginosa* regrow<br>- No change in susceptibility<br>- Change in susceptibility (S → R)*	15 (42.9%)<br>9 (25.7%)<br>6 (17.1%)

S = susceptible; R = resistant

Table S3. Logistic regression analysis of factors associated with treatment failure during OPAT

	***p* value**	**OR (IC95%)**
**Baseline Characteristics**		
Gender, male	**0.022**	3.649 (1.203-11.070)
Comorbidities		
COPD comorbidity	0.122	2.080 (0.822-5.264)
**Related to infection**		
Diagnosis		
Endovascular infection	0.195	2.878 (0.582-14.229)
**Treatment Characteristics**		
Antimicrobial treatment		
Monotherapy		
Meropenem	0.147	2.663 (0.708-10.013)
Other beta-lactams	0.153	3.250 (0.646-16.338)

OR: odds ratio; CI: confidence interval.

Table S4. Logistic regression analysis of factors associated with 30-day treatment failure

	p value	OR (IC95%)
**Baseline Characteristics**		
Age	**0.049**	1.022 (1.000-1.043)
Gender, male	0.089	1.804 (0.915-3.559)
Comorbidities		
Charlson score	**<0.001**	1.321 (1.132-1.540)
COPD comorbidity	0.140	1.634 (0.851-3.137)
Chronic heart failure	**0.013**	2.278 (1.190-4.358)
Malignancy	**0.002**	2.763 (1.441-5.298)
**Related to infection**		
Diagnosis		
Intra-abdominal or anorectal infection or abscess	**0.018**	4.250 (1.286-14.047)
Exacerbated COPD	**0.008**	3.919 (1.431-10.735)
Bronchiectasis exacerbation	0.111	0.570 (0.285-1.139)
Lung abscess	0.062	4.304 (0.930-19.922)
Endovascular infection	**0.011**	4.979 (1.451-17.089)
**Treatment Characteristics**		
Antimicrobial treatment		
Combination therapy (beta-lactams + aminoglycosides)	**0.012**	0.289 (0.110-0.761)
Monotherapy		
Other beta-lactams	0.211	2.429 (0.604-9.767)
Other non-beta-lactam antimicrobial	**0.049**	11.349 (1.007-127.914)

OR: odds ratio; CI: confidence interval.

Figure S1. Annual number of OPAT patients with *Pseudomonas aeruginosa* infection from 2012 to 2024.

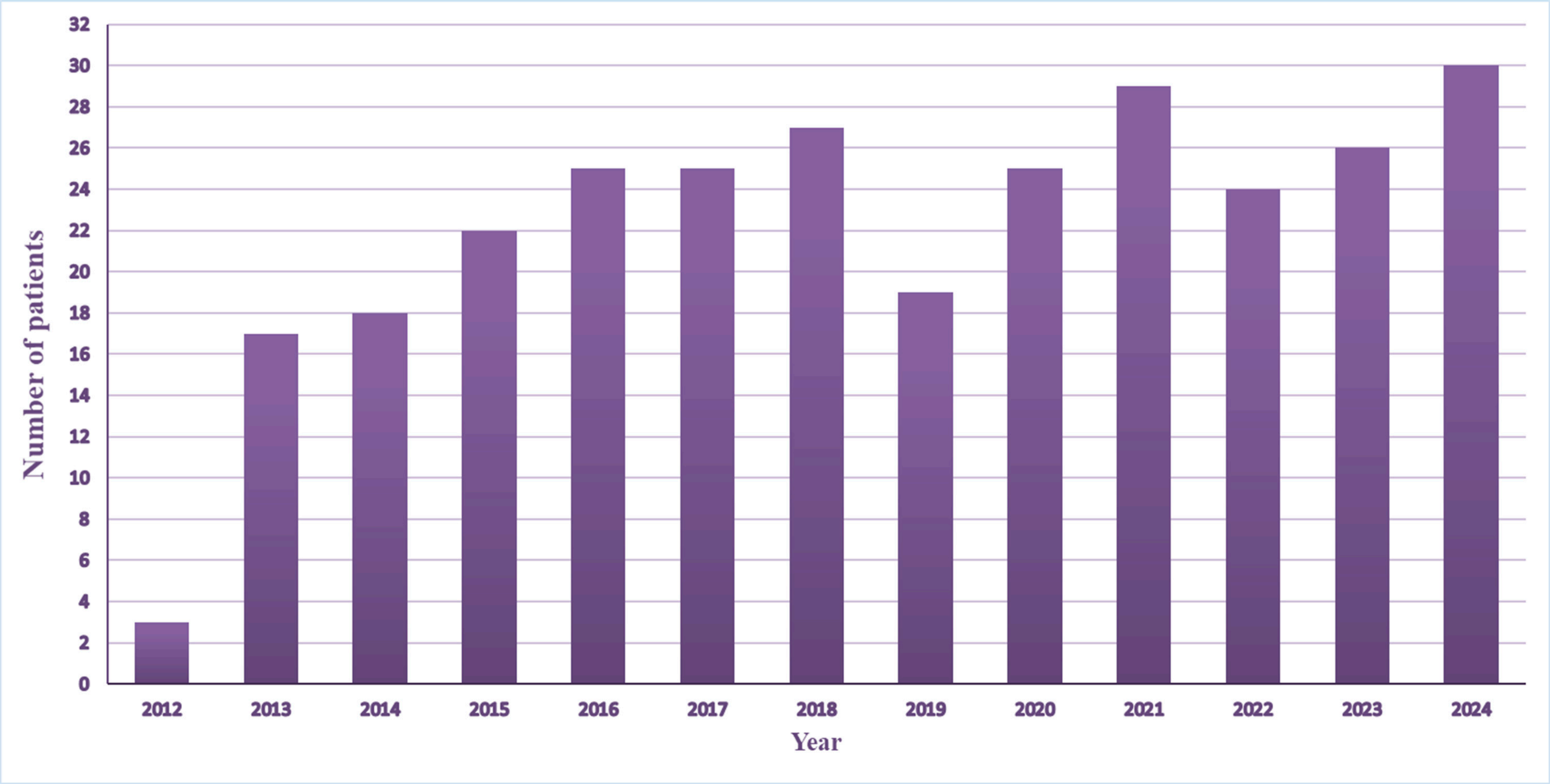

Supplement: Supplementary file 1 [file pharmaceutics-18-00549-s001.zip › pharmaceutics-4248213-supplementary.pdf]
